# Evaluation of the complexation behaviour among functionalized diphenyl viologens and cucurbit[7] and [8]urils

**DOI:** 10.1038/s41598-024-56370-1

**Published:** 2024-03-09

**Authors:** Bebin Ambrose, Gopal Sathyaraj, Murugavel Kathiresan

**Affiliations:** 1 Electro Organic and Materials Electrochemistry Division, CSIR-CECRI, Tamil Nadu, Karaikudi, 630 003 India; 2 Academy of Scientific and Innovative Research (AcSIR), Ghaziabad, 201002 India; 3 CLRI-CATERS, CSIR-Central Leather Research Institute, Tamil Nadu, Chennai, 600020 India

**Keywords:** Self-assembly, Chemical physics

## Abstract

The complexation behaviour of Diphenyl viologens (DPVs) with Cucurbit[n]urils (CB[n]) was evaluated in detail and the results were reported. In this work, we present the synthesis of various DPVs functionalised with electron withdrawing and electron donating groups (EWGs & EDGs) and investigate their complexation behaviour with CB[7] and CB [8]. Carboxylic acid functionalized DPV’s (DPV-COOH) complexation with CB[8] gives additional insights, i.e., indicates hydrogen bonding plays an effective role in the complexation. The formation of a 2:2 quaternary complex of DPV-COOH/CB[8] under neutral pH conditions was supported by various analytical techniques. The complexation of DPVs with CB[7] specifies that irrespective of the functional group attached, they all form a 1:2 ternary complex, but the findings elaborate that the pattern followed in the complexation depends on the EW or EDG attached to the DPVs. The competition experiments conducted between functionalized DPVs and CB[7], CB[8] shows that they have more affinity towards CB[8] than CB[7] because of the better macrocyclic confinement effect of CB[8], as confirmed using UV–Vis spectroscopy. The binding affinity among EWG and EDG functionalised DPVs with CB[8] concludes EDG functionalised DPVs show better affinity towards CB[8], because they can form a charge transfer complex inside the CB[8] cavity. Exploring these host–guest interactions in more complex biological or environmental settings and studying their impact on the functionality of DPVs could be an exciting avenue for future research.

## Introduction

Diphenyl viologens (DPVs) are an important structural motif in the viologen family and they can be synthesized via classical Zincke reaction in two steps^1–5^. The electron-donating groups (EDGs) and electron-withdrawing groups (EWGs) on the phenyl ring of the DPV backbone significantly influence its redox properties^6–8^. Also, the binding affinity and charge transfer (CT) complexation of the functionalized DPVs can be influenced by host molecules such as cyclodextrins (CDs), cucurbit[n]urils (CB[n]s), and calixarenes, etc., in the guest–host self-assembly^9–11^. Supramolecular chemistry is classically governed by the interaction between two or more entities that are not covalently linked but guided through intermolecular forces such as electrostatic forces, π-π stacking, hydrogen bonds, etc^12–15^. Interestingly, different complex molecular architectures can be designed based on guest–host assemblies and by the right selection of guest and host molecules, self-assembled architectures can be made. Therefore, choosing the right and most interactive partner becomes essential to design supramolecular self-assembly^16,17^.

CB[n]s are a water-soluble macrocyclic compound that has created a great interest in the field of supramolecular chemistry due to their ability to include various organic and bio-organic guest molecules like proteins, amino acids, peptides, etc., in their cavity^18–20^. The inner hydrophobic cavity of CB[n] provides favourable sites for the inclusion of guest molecules via non-covalent interactions. Moreover, the guests should possess a specific size and shape in order to enhance the selectivity and binding affinity towards cucurbiturils^21,22^. The construction of guest–host self-assemblies between viologen guests and cucurbituril hosts is an interesting and well-studied topic in the field of molecular self-assembly^18,23,24^. During this self-assembly/complexation, a viologen tends to donate an electron to the host CB[n], where it is enclosed inside the hydrophobic cavity of the latter. Thus, the system is stabilized by the CT complex and it may be considered the most recognized and challenging supramolecular binding force^25^. Furthermore, the complexation can be affected by external stimuli like pH, temperature, solvent polarity, and light which may cause the guest molecules to be released from the host under controlled conditions^26–28^. In this context, viologen-based CT molecular recognition with CB[8] has been broadly explored for the construction of viologen-based supramolecular frameworks or supramolecular polymers via viologen radical dimer (V^+•^…^•+^V) formation and their subsequent complexation inside the CB[8] cavity^29^. Our group has been constantly interested in the synthesis of different alkyl/aryl terminated viologens and their CT interactions with CB[8] for the construction of different supramolecules. There aren't many reports detailing the CT complexation with CB[8] host and related aryl viologen guest–host complexations. As a result, it is crucial to develop adaptable methods for comprehending the critical concept of CT interactions between aryl viologens and CB[8] based on optimising the electrochemical and optoelectronic characteristics of aryl viologen-based supramolecular systems.

**Figure 1:**
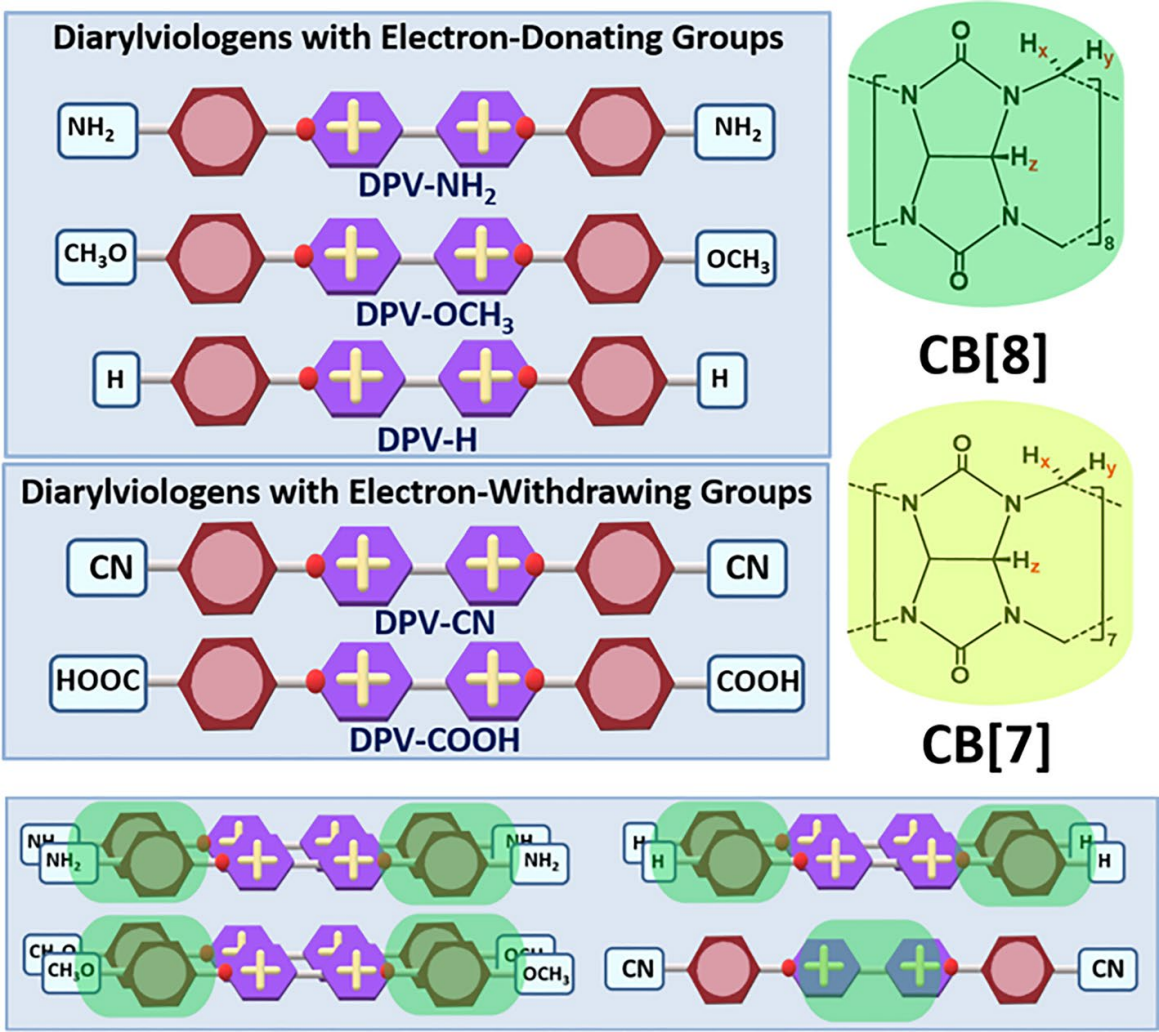
Schematic representation of viologens adopted and the cucurbiturils and their reported complexation behaviour with CB[8].

Scherman and co-workers investigated the binding mechanisms of various EWGs & EDGs substituted viologens with the CB[8] cavity^30^. The report described that the DPVs bearing electron-rich substituents on their backbone predominantly exhibited 2:2 binding stoichiometry, whereas, electron-deficient substitutions conversely formed a 1:1 binary complex. In their study, they demonstrated the spectroscopic behaviours of the closely packed aryl viologen guests via π–π stacking and CT radical dimer interactions inside the CB[8] cavity. Ni and coworkers conducted research solely on COOH-functionalized DPV and examined their interactions with CB[8] under conditions of pH 2.1^31^. They deduced that the guest molecules undergo linear J-type polymerization, wherein two terminal carboxylphenyl groups from the guest molecules are accommodated within the CB[8] cavity. The resulting supramolecular structure exhibits photochromic properties, making it a promising contender for intelligent photochromic materials. Despite being effective, progress in the design of new aryl viologens, particularly, EWGs-functionalized aryl viologens and their relative supramolecular interactions is highly demanded. In this work, we report the synthesis of five distinct DPVs functionalized with EWGs and EDGs (Fig. 1) and their different binding modes with CB[7] and CB[8] hosts. In particular, much attention was focused on DPVs functionalized with EDGs and their competitive binding nature with CB[7] and CB[8]. Besides, the competition in the binding modes between EDG and EWG functionalized DPVs with CB[8] was also investigated.

## Experimental

### Materials and reagents

4,4′-Bipyridine, *p*-diphenylamine, *p*-anisidine, 4-aminobenzonitrile, 4-aminobenzoic acid, glycoluril, and paraformaldehyde were procured from Alfa Aesar, India. 1-Chloro-2,4-dinitrobenzene, aniline, acetonitrile (ACN), tetrahydrofuran (THF), and ethanol were purchased from SRL Chemicals, India. All other reagents were of analytical grade and used as received and used without further purification. Water used in this experiment was doubly distilled over alkaline potassium permanganate using an all-glass apparatus.

### Characterization and measurements

Absorbance changes were measured with a Hewlett–Packard 8453 spectrophotometer. ^1^H NMR(Nuclear Magnetic Resonance), ^13^C NMR, DEPT (Distortionless Enhancement by Polarization Transfer), 2D-NOESY(Nuclear Overhauser Effect Spectroscopy) and DOSY (Diffusion Order Spectroscopy) spectra were recorded on a Bruker 500 Avance spectrometer at 25 °C using D_2_O as a solvent and internal reference. DLS (Dynamic Light Scattering) was carried out by a Nanotrac Ultra Particle size analyser. ESI–MS (Electron Spray Ionisation-Mass Spectrometry) was carried out by Waters Xevo TQD 2000 ESI instrument. Absorbance measurements were carried out in sodium phosphate buffer solution (0.1 M PBS).

### Synthesis

**Figure 2:**
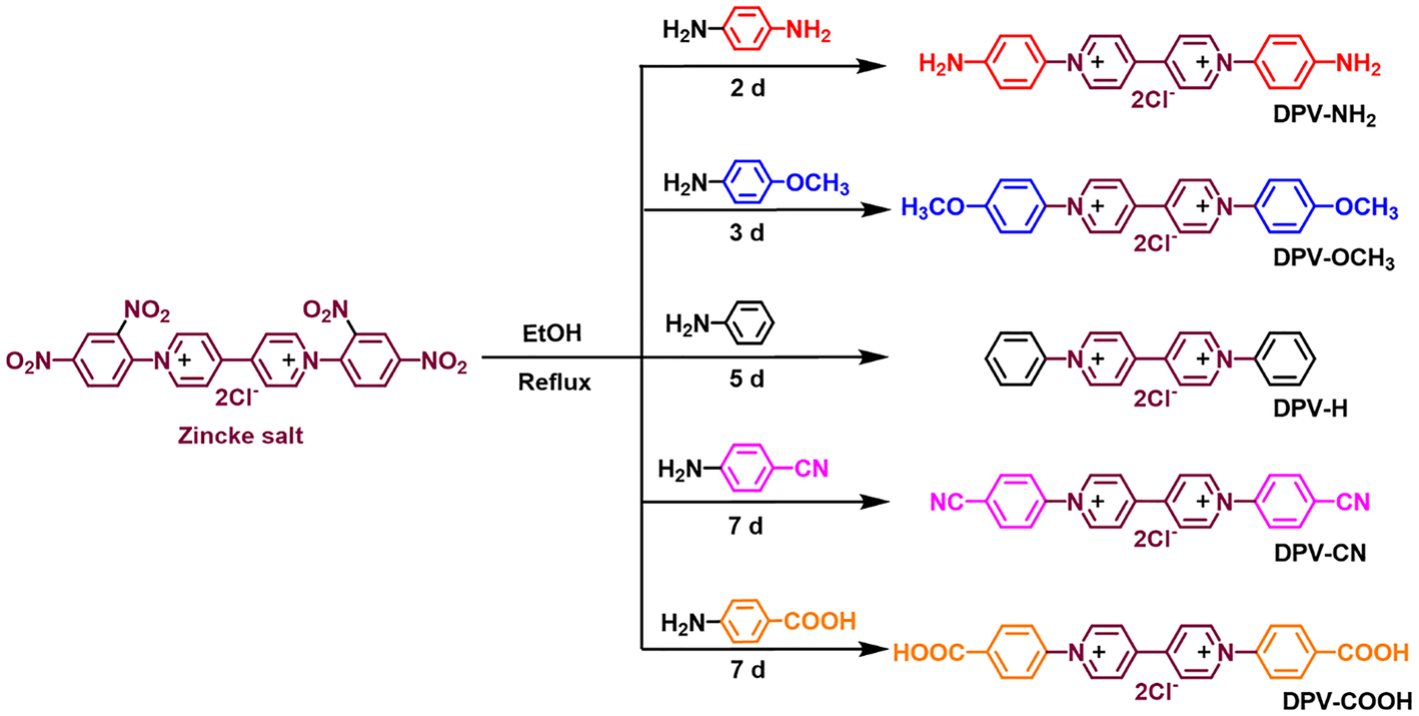
Synthetic routes of various DPVs.

All the DPVs were synthesized following reported procedures using the Zincke reaction (Fig. 2)^6^. The detailed synthetic procedure is given in the supporting information.

## Results and discussion

### Complexation behaviour of DPV-COOH with CB[8]

**Figure 3:**
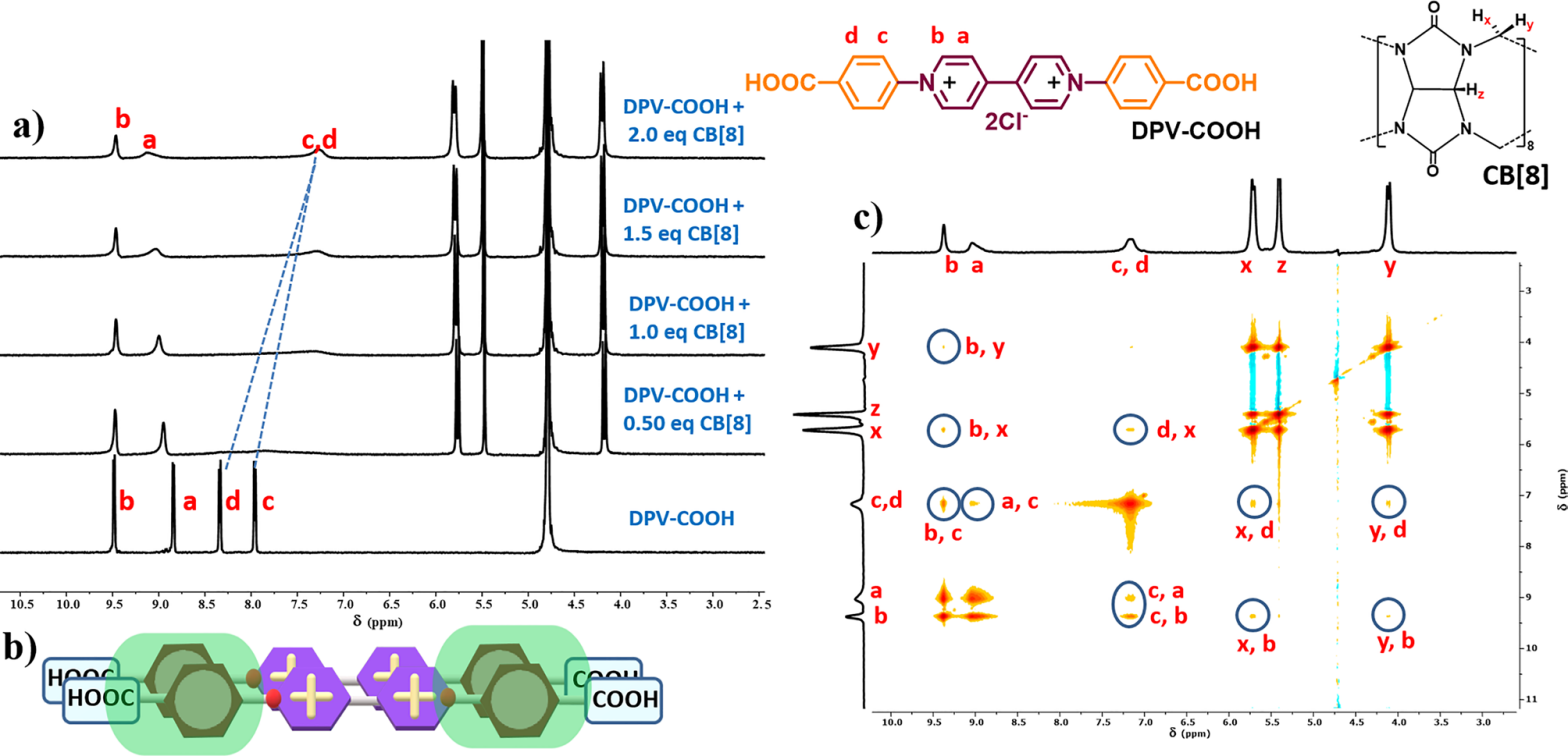
(**a**) ^1^H NMR spectra of DPV-COOH with variable concentrations of CB[8] (D_2_O). (**b**) Schematic representation of 2:2 quaternary complex. (**c**) 2D-NOESY correlation of DPV-COOH with 2.0 equiv. of CB[8].

Scherman and co-workers investigated the different binding modes of DPV-NH_2_, DPV-OCH_3_, DPV-H, DPV-CN, etc. with CB[8] and discovered that EDG and EWG functionalized viologens form 2:2 quaternary complexes and 1:1 binary complex respectively^30^. The same results were obtained in our investigations under identical conditions. In addition to the reported DPVs^30^, the binding nature of DPV-COOH was investigated in this work and it was found that it shows a distinct complexation behaviour resulting in the formation of a 2:2 quaternary complex. Therefore, we initiated a tried-and-true method to analyse the different binding natures of DPVs with CB[7] and CB[8] using the ^1^H NMR titration technique^32^. Formation of a 2:2 quaternary complex is confirmed by the ^1^H NMR titration of DPV-COOH with CB[8] (Fig. 3a), phenyl ring protons ‘c’ and ‘d’ of DPV-COOH experienced an upfield shift of 0.71 and 1.08 ppm, respectively, with the addition of 2 equiv. of CB[8]. This is a definite sign that the phenyl ring was included in the CB[8] cavity. It is further confirmed by the bipyridinium ‘a’ proton, which experienced a downfield shift of 0.15 ppm and revealed close packing of ‘a’ proton with a phenyl ring inside the CB[8] cavity. Additionally, formation of a 2:2 quaternary complex was confirmed by ESI–MS (Figure S1) and 2D-NOESY techniques. Notably, protons that are close to one another in space would show off-diagonal correlations in NOESY spectra. The DPV-COOH NOESY spectrum (Figure S2) displayed that the ‘c’ and ‘b’ protons had a significant correlation. The correlation spectrum of DPV-COOH + two equiv. of CB[8] (Fig. 3c) showed a strong correlation between ‘c’ and ‘a’ protons and substantiated how the bipyridinium units are stacked together within the CB[8] cavity. From the result obtained from the correlation spectra, one can assume that the DPV-COOH units are parallelly aligned, i.e., the units are likely to stack rather than sit exactly on top of one another, as illustrated in Fig. 3b. Further, the correlations of the ‘x’ and ‘y’ protons of CB[8] with the ‘b’ and ‘d’ protons of DPV-COOH additionally supported the idea that CB[8] was enclosed in the way described above. DOSY analysis also provides evidence for the formation of a 2:2 quaternary complex based on the measured diffusion coefficient (D) values. For DPV-COOH and DPV-COOH + 2 equiv. CB[8], the D values observed are 4.13 × 10^–10^ and 3.69 × 10^–10^, respectively (Figures S3 and S4). The minimal alteration in the D value upon the addition of CB[8] suggests that there is no significant supramolecular polymerization under neutral pH conditions.

With these results, the formation of a 2:2 quaternary complex in the DPV-COOH + 2.0 equiv. CB[8] system was established. DLS corroborates our results by demonstrating that the particle sizes for DPV-COOH, CB[8], and DPV-COOH + 2.0 equiv. CB[8] are 129 nm, 205 nm, and 223 nm respectively (Figure S5). The addition of 2.0 equiv. of CB[8] to DPV-COOH resulted in minimal change in the complex size, indicating the absence of supramolecular polymerization under neutral pH conditions which further complements DOSY results. This contrasts with Ni and coworkers’ observation of supramolecular polymerization at pH 2.1^31^. Our results indicate that the interaction between CB[8] and DPV-COOH is pH-dependent, forming a 2:2 quaternary complex at neutral pH. According to the literature reports CB[8] tends to form multiple hydrogen bonds with guest molecules that are functionalized with amines(NH_2_), hydroxyls (OH), and acids (COOH)^33,34^. Among them, the acid (COOH) group has a higher ability to create a hydrogen bond with the carbonyl gateway of CB[8], which might be the reason, the system gets 2:2 binding stoichiometry. NMR titration studies additionally supported the formation of hydrogen bonds; typically, the exchange of bound CB[8] with an unbound one results in the splitting of CB[8] peaks in the 2:2 quaternary complex produced by EDG functionalized DPVs. The CB[8] proton signals were not split in Fig. 3a further signifying that the formation of multiple hydrogen bonds that may have limited the exchange of CB[8] host molecules.

### Complexation behaviour of DPVs with CB[7]

**Figure 4:**
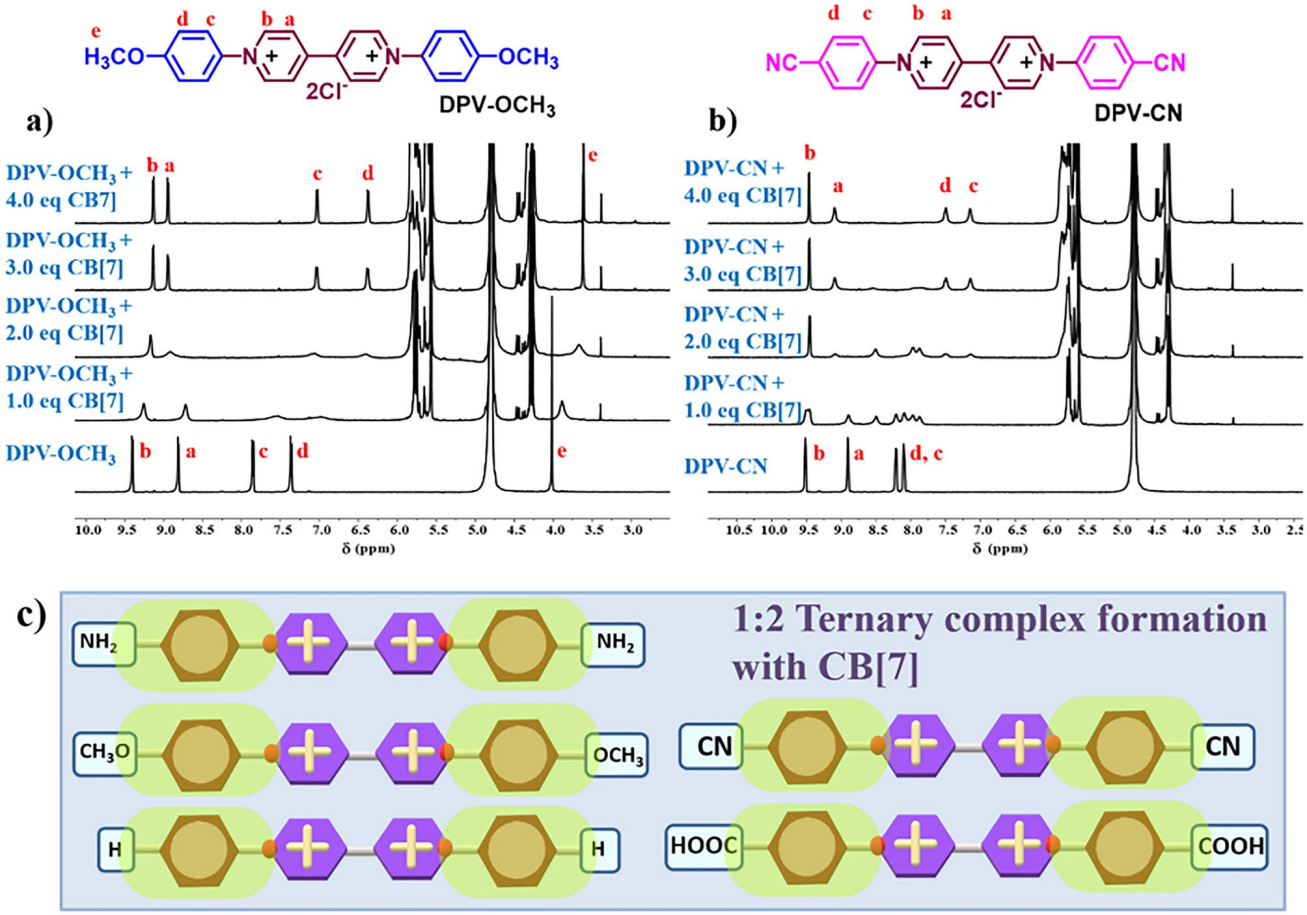
(**a**) ^1^H NMR spectra of DPV-OCH_3_ with variable concentrations of CB[7] (D_2_O). (**b**) ^1^H NMR spectra of DPV-CN with variable concentrations of CB[7] (D_2_O). (**c**) Schematic representation of 1:2 ternary complexation with all the five DPVs.

The complexation behaviour of all the as-synthesized DPVs was investigated using ^1^H NMR spectroscopic technique. All the guest molecules (DPVs) showed a 1:2 ternary binding stoichiometry with CB[7] irrespective of their electronic nature (EDG or EWD). Initially, ^1^H NMR titration of DPV-OCH_3_ in Fig. 4a revealed that the phenyl ring protons ‘c’ and ‘d’ were shifted upfield by 0.74 and 0.99 ppm, respectively, indicating that the phenyl ring was encapsulated into the hydrophobic cavity of CB[7] host. Moreover, the upfield shift of the bipyridinium ‘b’ proton by 0.26 ppm represented the closer arrangement of CB[7] to the bipyridinium core inside the cavity. Similarly, changes in chemical shift values were observed continuously for the further increase in the concentration of CB[7] to bring the as-formed 1:2 ternary complex closer. Later, the nature of binding modes was examined for DPV-NH_2,_ and a similar NMR signal pattern was seen as in previous guest DPV-OCH_3_, which adopted the formation of a 1:2 ternary complex with CB[7] (Figure S6). The phenyl protons ‘c’, ‘d’, and ‘e’ showed a large upfield shift of 0.7 ppm, whereas the viologen ‘b’ protons displayed a lower upfield shift of 0.27 ppm in DPV-H. The shifts in ^1^H NMR signals correspond to (Figure S7) 1:2 ternary complex formation in DPV-H. In a similar fashion, EWG substituted DPVs experienced 1:2 ternary complexation but a different ^1^H NMR pattern was observed. When one equiv. of CB[7] was added to EWG functionalized DPVs, the ‘a’ proton shifted to the upfield side until the addition of two equiv. of CB[7] which indicates the inclusion of bipyridinium moiety inside the CB[7], and as a result, initially 1:1 binary complex is formed. Then a downfield shift of ‘a’ was observed after the addition of the third equivalent of CB[7] which indicates the formation of a 1:2 ternary complex. When three equiv. of CB[7] were added to DPV-CN guest, the following chemical shifts were observed; the bipyridinium proton ‘a’ experienced an upfield shift of 0.41 ppm, whereas protons in the phenyl ring ‘c’ and ‘d’ experienced upfield shifts of 0.96 and 0.72 ppm, respectively (Fig. 4b). Finally, in the DPV-COOH + CB[7] guest–host system, a similar ^1^H NMR pattern was detected and both the systems formed 1:2 ternary complexes (Figure S8).

Specifically, the EDG modified DPVs exhibit a 1:2 ternary complexation with CB[7] right from the initial addition, showcasing swift exchange between two CB[7] molecules. This phenomenon is confirmed by the splitting of ^1^H NMR signals of CB[7] protons. In contrast, EWG modified DPVs, DPV-COOH or DPV-CN, also form a 1:2 ternary complex with CB[7]. However, upon the first addition of CB[7] to either DPV-COOH or DPV-CN, a 1:1 binary complex is initially formed, encapsulating the bipyridyl moiety. Upon adding a third equiv. of CB[7], the system transitions into the 1:2 ternary complexation. Like the EDG scenario, rapid shuttling of two CB[7] equivalents is evidenced by the splitting of ^1^H NMR signals of the CB[7] protons. The complexation behaviour of DPV-CN is elucidated in Figure S9, emphasizing that the interaction initiates with the addition of 0.25 equiv. of CB[7]. This study emphasises that the interaction patterns between DPVs and CB[7] differ based on their functional groups attached even though they all form a 1:2 ternary complex **(**Fig. 4c).

### Competition experiments of DPVs with CB[7] and CB[8]

**Figure 5:**
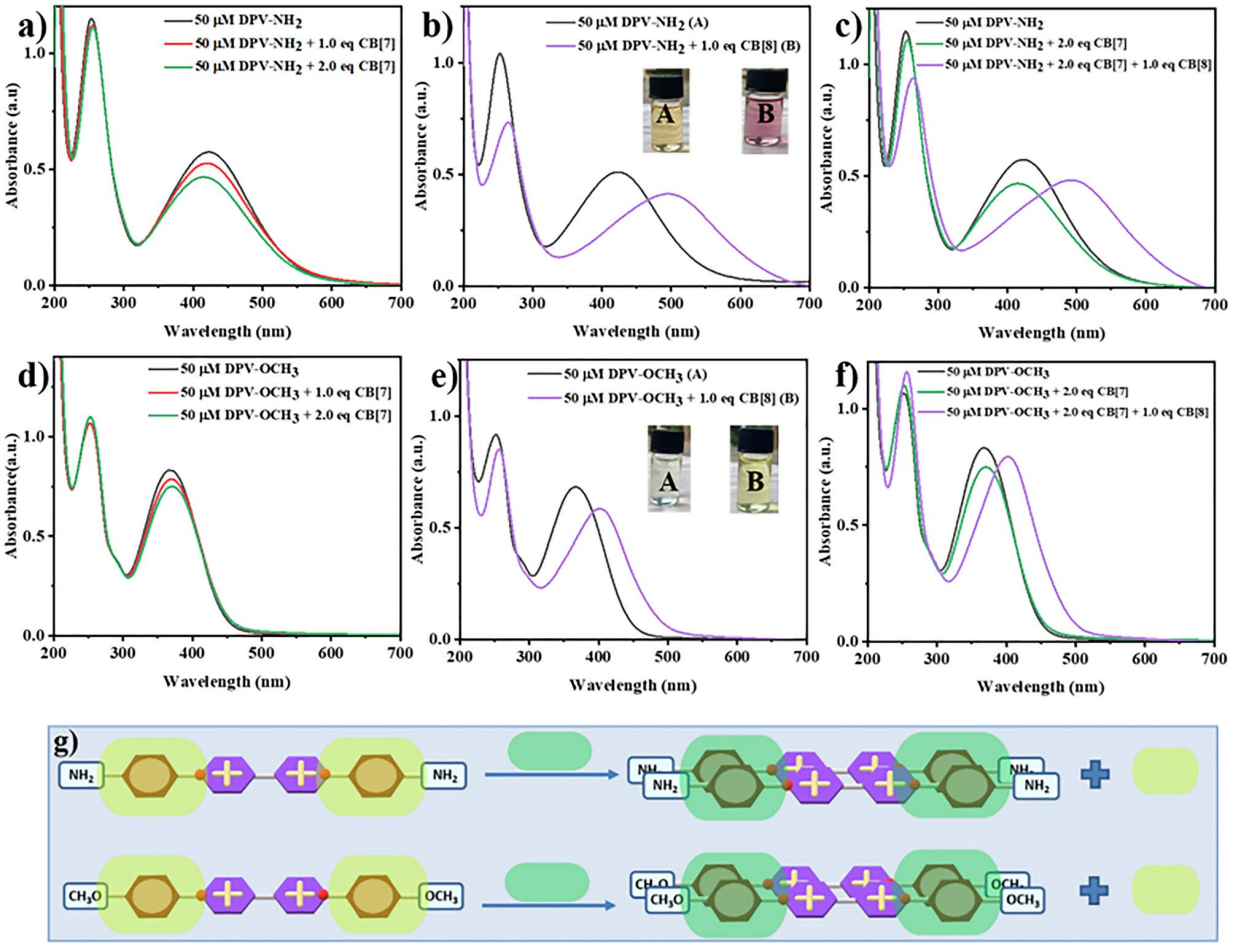
UV–Vis absorbance spectra of DPV-NH_2_ with (**a**) CB[7], (**b**) CB[8] and (**c**) with CB[7] and CB[8]. UV–Vis absorbance spectra of DPV-OCH_3_ with (**d**) CB[7], (**e**) CB[8] and (**f**) with CB[7] and CB[8]. (**g**) Schematic representation of the EDG functionalized DPVs complexation with CB[7] and CB[8].

The competition studies were performed to investigate the binding modes of DPVs with CB[7] and CB[8] using UV–visible spectroscopy in 0.1 M PBS^35^. Studies were initiated with the DPV-NH_2_ viologen guest. At first, two equiv. of CB[7] was added to the DPV-NH_2_ solution, and a decrease in the absorbance maxima (hypochromic shift) was noted indicating the encapsulation of phenyl rings of DPV-NH_2_ into the CB[7] cavity (formation of a 1:2 ternary complex) (Fig. 5a). Then, one equiv. of CB[8] was added into the DPV-NH_2,_ the absorption maxima shifted from 422 to 499 nm (redshift), and a visible colour change from pale brown to purple was noted, due to CT interactions between CB[8] and DPV-NH_2_ upon 2:2 quaternary complexation through host–guest self-assembly (Fig. 5b). It is important to mention that, when CB[8] is introduced into the solution containing the DPV-NH_2_/CB[7] ternary complex, it can also interact with both the viologen and CB[7] molecules independently. Due to the larger cavity size of CB[8], small molecules (bipyridinium, phenyl ring) may not perfectly fit into its cavity. Therefore, after 1.0 equiv. addition of CB[8] into the ternary complex (DPV-NH_2_ + 2CB[7]) solution, it may occupy the sites that already have been occupied by CB[7] by replacing CB[8] host molecules are observed by the red shift in UV–Vis spectra and purple colour change (Fig. 5c). In order to form a stable complex, it can accommodate one more DPV-NH_2_ unit (due to the large cavity size) and create a stable 2:2 quaternary complex. Similarly, identical UV–Vis spectroscopic investigations were carried out for DPV-OCH_3_. At first, two equiv. of CB[7] was added to the DPV-OCH_3_ solution, and a hypochromic shift was noted as earlier indicating the formation of a 1:2 ternary complex (Fig. 5d). It was noticed that upon the addition of one equiv. of CB[8] to DPV-OCH_3,_ a red shift with absorption maxima ranging from 368 to 402 nm was observed and a colour change from transparent to yellow was detected (Fig. 5e) which confirmed the formation of a 2:2 quaternary complex. When 1 equiv. of CB[8] was added to the ternary complex of DPV-OCH_3_ + 2CB[7], a red shift in UV–Vis spectra and transparent to pale yellow colour changes were observed indicative of a greater affinity of CB[8] resulting in the formation of a stable 2:2 quaternary complex (Fig. 5f). With these, we desired to compare the complexation ability of DPVs (DPV-NH_2_ and DPV-OCH_3_) with CB[7] and CB[8]. In this aspect, both CB[7] and CB[8] were added to the DPV-NH_2_ and DPV-OCH_3_ solutions separately, and their complexation behaviours were investigated. These studies revealed that both functionalized DPVs formed 2:2 quaternary CT complexes (Fig. 5c and f) in a similar fashion. The red shift and colour changes were noticed when CB[8] was added to the solution containing 2CB[7] + DPVs (DPV-NH_2_ or DPV-OCH_3_) indicating that the DPVs have a higher propensity to form a complex with CB[8] (Fig. 5g) due to preferential CT complexing behaviour.

**Figure 6:**
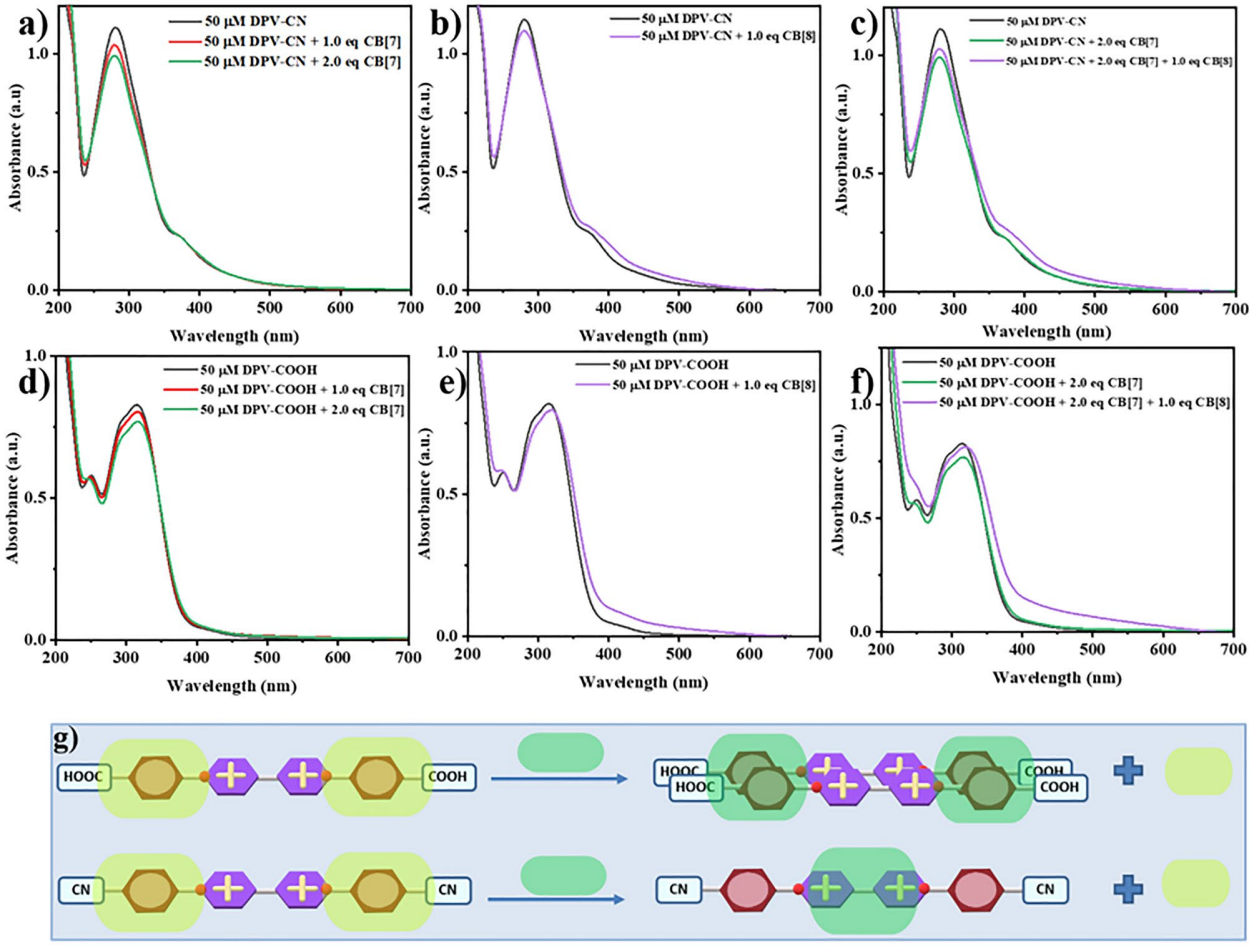
UV–Vis absorbance spectra of DPV-CN with (**a**) CB[7], (**b**) CB[8], and (**c**) with CB[7] and CB[8]. UV–Vis absorbance spectra of DPV-COOH with (**d**) CB[7], (**e**) CB[8], and (**f**) with CB[7] and CB[8]. (**g**) Schematic representation of the EWG functionalized DPVs complexation with CB[7] and CB[8].

In the sequence of investigations, the next UV–Vis spectroscopic studies involved the evaluation of the binding nature of EWG functionalized DPVs (DPV-COOH & DPV-CN). Figure 6a shows the decrease in absorbance maxima when two equiv. CB[7] was added to the DPV-CN solution which confirmed the 1:2 ternary complexation. Figure 6b shows that when one equiv. of CB[8] was added to DPV-CN, there was a slight decrease in the intensity of absorbance without any shift in the wavelength and these observations clearly denoted the formation of a 1:1 binary complex^30^. When one equiv. of CB[8] was added to the ternary complex (DPV-CN + 2 equiv. of CB[7]), there was a slight increase in absorbance as well the pattern resembled the pattern of DPV-CN + one equiv. of CB[8] indicating that DPV-CN also has a greater affinity towards CB[8] and forms 1:1 binary complex (Fig. 6c). Likewise, to investigate the binding nature of DPV-COOH guest, 1:2 ternary complex of DPV-COOH and 2 equiv. CB[7] was confirmed by the hypsochromic shift as in earlier results (Fig. 6d). Further, the addition of 1 equiv. of CB[8] to DPV-COOH resulted in the shift of absorption maxima from 315 to 320 nm signifying a 2:2 quaternary complex formation and these results complemented the ^1^H NMR titration results(Fig. 6e). It is significant to mention that considerable red shift and colour changes were seen when 1 equiv. of CB[8] was introduced to DPV-NH_2_ and DPV-OCH_3_ (Fig. 5b and e). However, in the case of DPV-COOH, no colour change was noticed upon complexation, and the changes in the absorbance maxima values are only about 5 nm. This result suggested the absence of CT complexation between the CB[8] inner cavity and the viologen units due to the electron-accepting tendency of the COOH group may arrest the flow of electrons between the stacked rings inside the cavity. The competition results of DPV-COOH (Fig. 6f) resembled the results of DPV-EDG with CB[7] and [8] indicating that all these substituted DPVs have more affinity towards CB[8] than CB[7] and form a stable 2:2 quaternary complex, whereas DPV-CN formed a stable 1:1 binary complex.

The results based on the absorbance patterns in the competition studies indicate that the DPVs exhibited a stronger affinity for CB[8] than CB[7], irrespective of the formation of a 2:2 CT complex/2:2 quaternary complex or 1:1 binary complex(Fig. 6g). The factors such as larger size, improved shape complementarity, enhanced hydrophobic interactions and better macrocyclic confinement effects in CB[8] compared to CB[7] may result in a higher affinity for aryl viologens towards CB[8]^36–38^.

### Competition experiments of EWG and EDG functionalized DPVs with CB[8]

**Figure 7:**
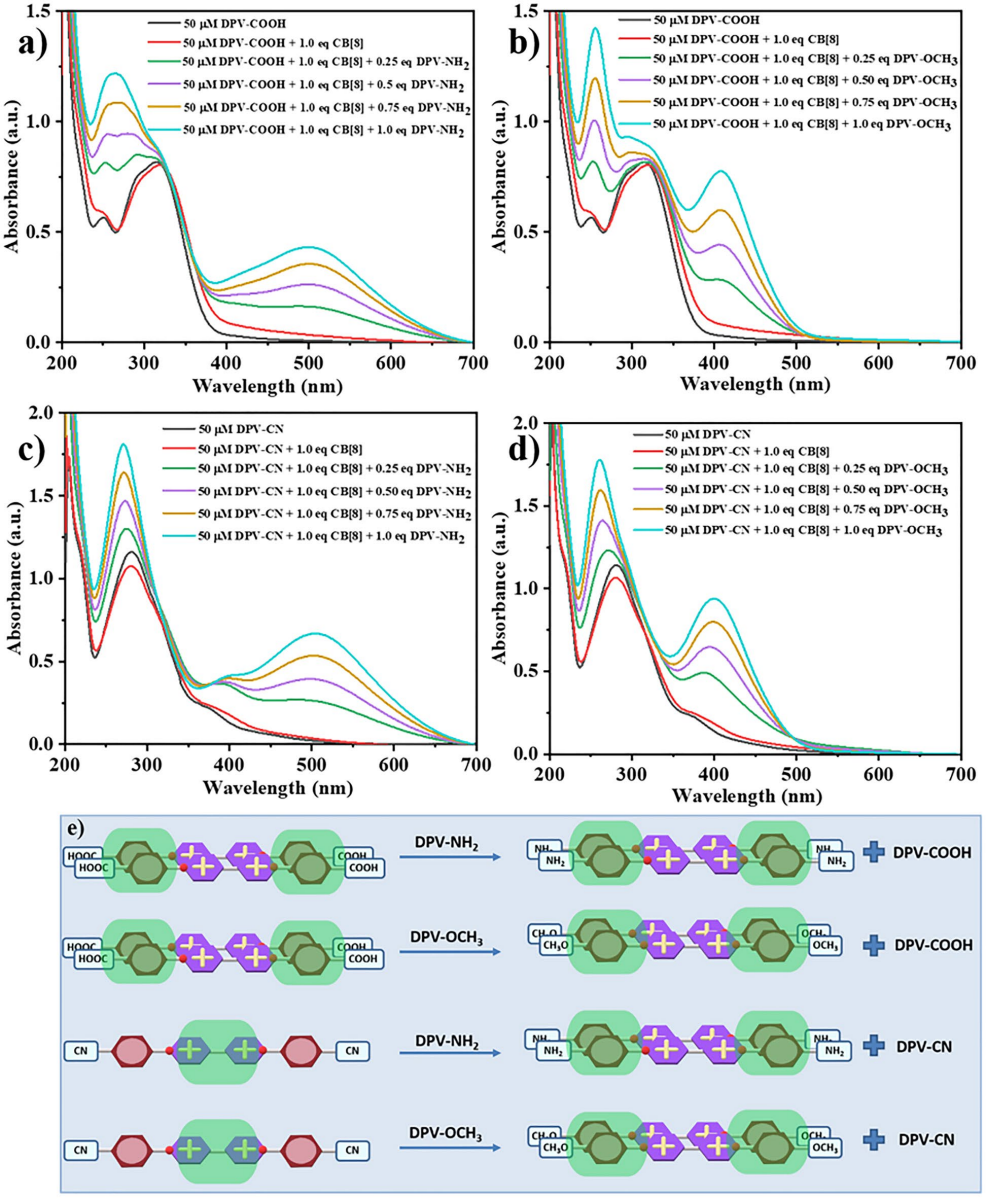
UV–Vis absorbance spectra of DPV-COOH with one equiv. of CB[8] and various concentrations of (**a**) DPV-NH_2_ and (**b**) DPV-OCH_3_ respectively; UV–Vis absorbance spectra of DPV-CN with one equiv. of CB[8] and various concentrations of (**c**) DPV-NH_2_ and (**d**) DPV-OCH_3_ respectively; (**e**) Schematic representation of the complexation competition of EWG functionalized DPV with EDG functionalized DPV.

To further understand the binding ability of EWG and EDG functionalized DPVs with CB[8], a competition experiment was conducted with UV–Vis spectroscopy. In the first experiment, the competition between the 2:2 quaternary complex of DPV-COOH/CB[8] and DPV-NH_2_ was examined (Fig. 7a). A small increment of 5 nm in the absorption maxima was identified after recording the absorbance spectra of DPV-COOH with one equiv. of CB[8]. Then 0.25 equiv. of DPV-NH_2_ was added subsequently to the same solution, and a good enhancement in the absorbance maxima at 500 nm was detected due to the encapsulation of DPV-NH_2_ into the CB[8] cavity. The colour change from pale yellow to a purple hue additionally supported the formation of a 2:2 CT complex with DPV-NH_2_. Likewise, when DPV-OCH_3_ was added to the DPV-COOH/CB[8] complexed solution, an absorbance maxima peak at 402 nm and a yellow hue colour were detected, showing that CB[8] preferentially encapsulated DPV-OCH_3_ due to its strong affinity nature towards CB[8] (Fig. 7b). Similar results were obtained when the experiments were conducted using DPV-NH_2_ and DPV-OCH_3_ guests to DPV-CN/CB[8] 1:1 binary complex. Initially, the guests were added to the DPV-CN/CB[8] 1:1 binary complex separately, the formation of a 2:2 CT complex with DPV-NH_2_ and DPV-OCH_3_ were observed, which are shown in Fig. 7c and d with respective absorbance maxima at 500 nm and 402 nm respectively.

The question arises here whether the DPVs form a homodimer inside the cavity. However, the observations from UV–Vis spectroscopy in this scenario support the formation of a 2:2 homoquaternary complex involving DPV-NH_2_ or DPV-OCH_3_ with CB[8], along with DPV-COOH or DPV-CN. Upon adding DPV-NH_2_ or DPV-OCH_3_ to DPV-COOH or DPV-CN + CB[8], distinct peaks emerged at 500 nm accompanied by a purple coloration, and at 402 nm along with a yellow coloration, indicating the formation of a 2:2 homoquaternary CT complexes. Overall, UV–Vis spectral studies revealed that the CB[8] has a stronger propensity to form 2:2 CT complexes, which are schematically depicted in Fig. 7e. Notably, the introduction of positive charge densities by staking two bipyridinium units of DPVs inside the CB[8] cavity can induce efficient electrostatic interactions with the negatively charged portal of CB[8]. This interaction increases the affinity of EDG-functionalized DPVs toward CB[8].

## Conclusions

This study investigates the interaction of functionalized DPVs with CB[7] and CB[8] under neutral pH conditions. Further, we have systematically analysed the complexing ability of DPV-COOH with CB[8] and examined the binding behaviour of functionalized DPVs (EWG and EDG groups) with CB[7] at neutral pH. The results show that DPV-COOH forms a 2:2 quaternary complex under neutral pH, supported by ^1^H NMR, 2D-NOESY, 2D-DOSY and DLS. This complex formation is attributed to the multiple hydrogen bonds between COOH and CB[8]. All DPVs form a 1:2 ternary complex with CB[7], but the complexation pattern differs for EWG and EDG functionalized DPVs. EDG functionalized DPVs form a 1:2 complex from the outset, while EWG functionalized DPVs initially form a 1:1 complex, transitioning to a 1:2 complex on the third equivalent addition of CB[7]. In this novel approach, competition experiments were conducted between DPVs and CB[7] and CB[8], revealing that, regardless of the functional group, all DPVs exhibit a higher affinity for CB[8] due to its larger cavity size, enhanced hydrophobic interactions, and improved macrocyclic confinement effects. Additionally, competition experiments between EDG and EWG functionalized DPVs with CB[8] indicate a higher affinity of EDG functionalized DPVs, attributed to the formation of charge-transfer complexes and better orbital overlap within the cavity. This understanding of DPV interactions with CB[7] and CB[8] lays the foundation for potential applications in drug delivery, sensing, or molecular recognition processes. Additionally, tailoring the molecular structure of DPVs to optimize their binding affinity and selectivity for specific CB[n]uril hosts could enhance their potential applications and broaden the scope of supramolecular chemistry.

### Supplementary Information

Supplementary Information.

## Data Availability

The datasets used and/or analysed during the current study available from the corresponding author on reasonable request.
