# Supplementary material for: Evaluation of the complexation behaviour among functionalized diphenyl viologens and cucurbit[7] and [8]urils
Source: Sci Rep. 2024 Mar 9;14:5786. doi: 10.1038/s41598-024-56370-1 (PMC10924918; doi:10.1038/s41598-024-56370-1)
Supplement: Supplementary file 1 — Supplementary Information. [file 41598_2024_56370_MOESM1_ESM.docx]

**Supplementary Information**

**Evaluation of the complexation behaviour among functionalized diphenyl viologens and cucurbit[7] and [8]urils**

Bebin Ambrose,^a,b^ Gopal Sathyaraj^c^, Murugavel Kathiresan^a,b^*

^a^Electro organic and Materials Electrochemistry Division, CSIR-CECRI, Karaikudi-630 003, Tamil Nadu, India. E-mail: kathiresan@cecri.res.in

^b^Academy of Scientific and Innovative Research (AcSIR), Ghaziabad-201002, India.

^c^ CLRI-CATERS, CSIR-Central Leather Research Institute, Chennai - 600020, Tamilnadu, India.

1. **Synthesis of Zincke salt and DPVs**
   1. **Synthesis of 1,1'-Bis(2,4-dinitrophenyl)-[4,4'-bipyridine]-1,1'-diium dichloride (Zincke salt)**

4,4ʹ-Bipyridine (1.0 g, 6.4 mmol) was dissolved in 100 ml CH_3_CN and stirred at 80 °C. To this solution 10 equivalents of 1-Chloro 2,4-dinitrobenzene was added periodically for 5 days and stirred at 80^o^C. The progress of the reaction was monitored by TLC (MeOH: HOAc: H_2_O –10:4:1). After the completion of the reaction the reaction mixture was cooled to room temperature, after which the precipitate was filtered, washed three times with CH_3_CN (20 ml) and dried under reduced pressure to yield pale white solid (2.40 g, 66.8%).^1^H NMR (500 MHz, D2O) δ 9.43 – 9.38 (m, 4H), 9.34 (d, J = 2.4 Hz, 2H), 8.90 – 8.82 (m, 6H), 8.23 (d, J = 8.6 Hz, 2H). ^13^C NMR (126 MHz, D_2_O) δ 152.63, 149.85, 146.83, 142.80, 138.22, 131.08, 130.71, 127.52, 122.78.

**
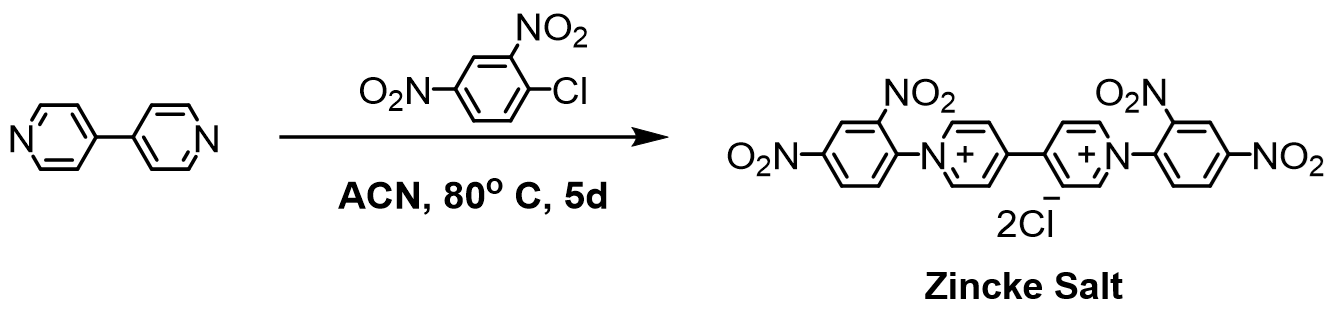
**

- 1. **Synthesis of** **1,1'-bis(4-aminophenyl)-[4,4'-bipyridine]-1,1'-diium dichloride (DPV-NH_2_)**

Zincke salt (0.5 g, 0.89 mmol) and p-Phenylenediamine (0.288 g, 2.67 mmol) were dissolved in 250 ml of ethanol. At 80 °C, the reaction mixture was stirred. TLC was used to track the reaction's progress (MeOH: HOAc: H_2_O - 10:4:1). The reaction mixture was stirred for a total of two days. After cooling to room temperature, the reaction mixture was decreased to 100 ml under reduced pressure. Following the addition of 750 ml THF, the mixture was allowed to rest for 2 hours and then the precipitate was filtered and dried to yield a black solid. (0.3 g, 81.9 %). ^1^H NMR (500 MHz, D2O) δ 9.19 (d, J = 6.4 Hz, 4H), 8.60 (d, J = 6.5 Hz, 4H), 7.51 (d, J = 8.4 Hz, 4H), 6.96 (d, J = 8.4 Hz, 4H). ^13^C NMR (126 MHz, D2O) δ 150.30, 149.21, 144.57, 133.42, 126.67, 125.04, 116.49.

**1.3 Synthesis of 1,1'-bis(4-methoxyphenyl)-[4,4'-bipyridine]-1,1'-diium dichloride (DPV-OCH_3_)**

Zincke salt (0.5 g, 0.89 mmol) and p-Anisidine (0.328 g, 2.67 mmol) were dissolved in 250 ml of ethanol. At 80 °C, the reaction mixture was stirred. TLC was used to track the reaction's progress (MeOH: HOAc: H_2_O - 10:4:1). The reaction mixture was stirred for a total of three days. After cooling to room temperature, the reaction mixture was decreased to 100 ml under reduced pressure. Following the addition of 750 ml THF, the mixture was allowed to rest for 2 hours and then the precipitate was filtered and dried to yield a brownish yellow solid. (0.320 g, 81.6 %). ^1^H NMR (500 MHz, D2O) δ 9.14 (d, J = 6.4 Hz, 4H), 8.54 (d, J = 6.5 Hz, 4H), 7.58 (d, J = 8.7 Hz, 4H), 7.10 (d, J = 8.8 Hz, 4H), 3.75 (s, 6H). ^13^C NMR (126 MHz, D2O) δ 161.67, 149.92, 145.18, 135.50, 126.88, 125.51, 115.72, 55.91.

**1.4 Synthesis of 1,1'-diphenyl-[4,4'-bipyridine]-1,1'-diium chloride (DPV-H)**

Zincke salt (0.5 g, 0.89 mmol) and aniline (0.248 g, 2.67 mmol) were dissolved in 250 ml of ethanol. At 80 °C, the reaction mixture was stirred. TLC was used to track the reaction's progress (MeOH: HOAc: H_2_O - 10:4:1). The reaction mixture was stirred for a total of five days. After cooling to room temperature, the reaction mixture was decreased to 100 ml under reduced pressure. Following the addition of 750 ml THF, the mixture was allowed to rest for 2 hours and then the precipitate was filtered and dried to yield a pale brown solid. (0.320 g, 81.6 %). ^1^H NMR (500 MHz, D2O) δ 9.34 (d, J = 6.6 Hz, 4H), 8.72 (d, J = 6.6 Hz, 4H), 7.79 – 7.66 (m, 10H). ^13^C NMR (126 MHz, D2O) δ 150.54, 145.51, 142.22, 132.03, 130.64, 127.05, 124.05.

**1.5 Synthesis of 1,1'-bis(4-cyanophenyl)-[4,4'-bipyridine]-1,1'-diium chloride (DPV-CN)**

Zincke salt (0.5 g, 0.89 mmol) and 4-aminobenzonitrile (0.315 g, 2.67 mmol) were dissolved in 250 ml of ethanol. At 80 °C, the reaction mixture was stirred. TLC was used to track the reaction's progress (MeOH: HOAc: H_2_O - 10:4:1). The reaction mixture was stirred for a total of seven days. After cooling to room temperature, the reaction mixture was decreased to 100 ml under reduced pressure. Following the addition of 750 ml THF, the mixture was allowed to rest for 2 hours and then the precipitate was filtered and dried to yield a greyish green solid. (0.246 g, 64.2 %). ^1^H NMR (500 MHz, D2O) δ 9.41 (d, J = 5.3 Hz, 4H), 8.78 (d, J = 5.6 Hz, 4H), 8.11 (d, J = 9.0 Hz, 4H), 7.98 (d, J = 8.9 Hz, 4H). ^13^C NMR (126 MHz, D2O) δ 151.29, 145.64, 145.08, 134.92, 127.38, 125.39, 117.76, 115.18.

**1.6 Synthesis of 1,1'-bis(4-carboxyphenyl)-[4,4'-bipyridine]-1,1'-diium chloride (DPV-COOH)**

Zincke salt (0.5 g, 0.89 mmol) and 4- aminobenzoic acid (0.315 g, 2.67 mmol) were dissolved in 250 ml of ethanol. At 80 °C, the reaction mixture was stirred. TLC was used to track the reaction's progress (MeOH: HOAc: H_2_O - 10:4:1). The reaction mixture was stirred for a total of seven days. After cooling to room temperature, the reaction mixture was decreased to 100 ml under reduced pressure. Following the addition of 750 ml THF, the mixture was allowed to rest for 2 hours and then the precipitate was filtered and dried to yield a greyish green solid. (0.407 g, 97.6 %). ^1^H NMR (500 MHz, D2O) δ 9.41 (d, J = 6.4 Hz, 4H), 8.77 (d, J = 6.4 Hz, 4H), 8.28 (d, J = 8.3 Hz, 4H), 7.89 (d, J = 8.3 Hz, 4H). ^13^C NMR (126 MHz, D2O) δ 169.41, 151.01, 145.56, 145.12, 134.22, 131.76, 127.23, 124.57.


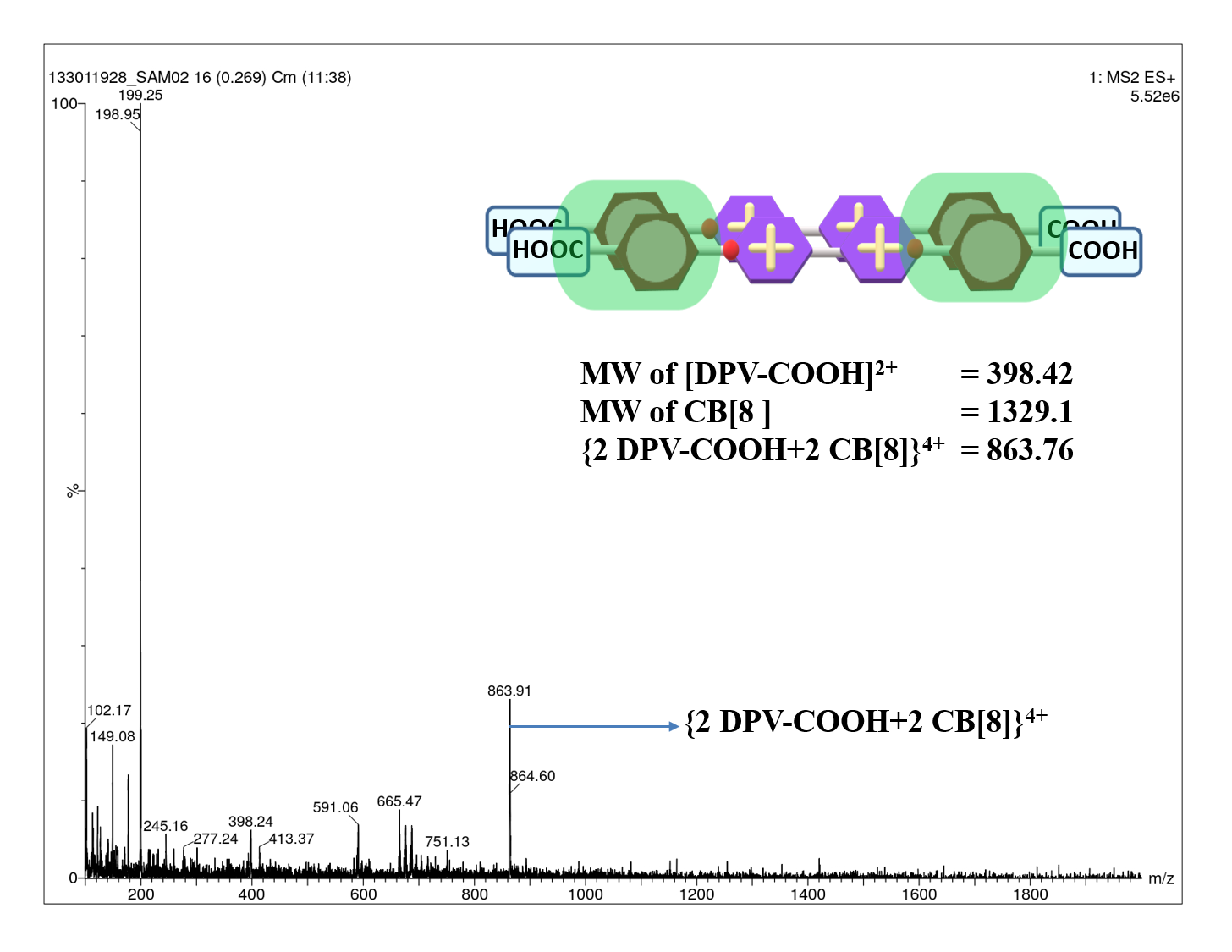
Figure S1: ESI-MS spectrum of 0.5 mM DPV-COOH and 1 mM CB[8] in aqueous solution.


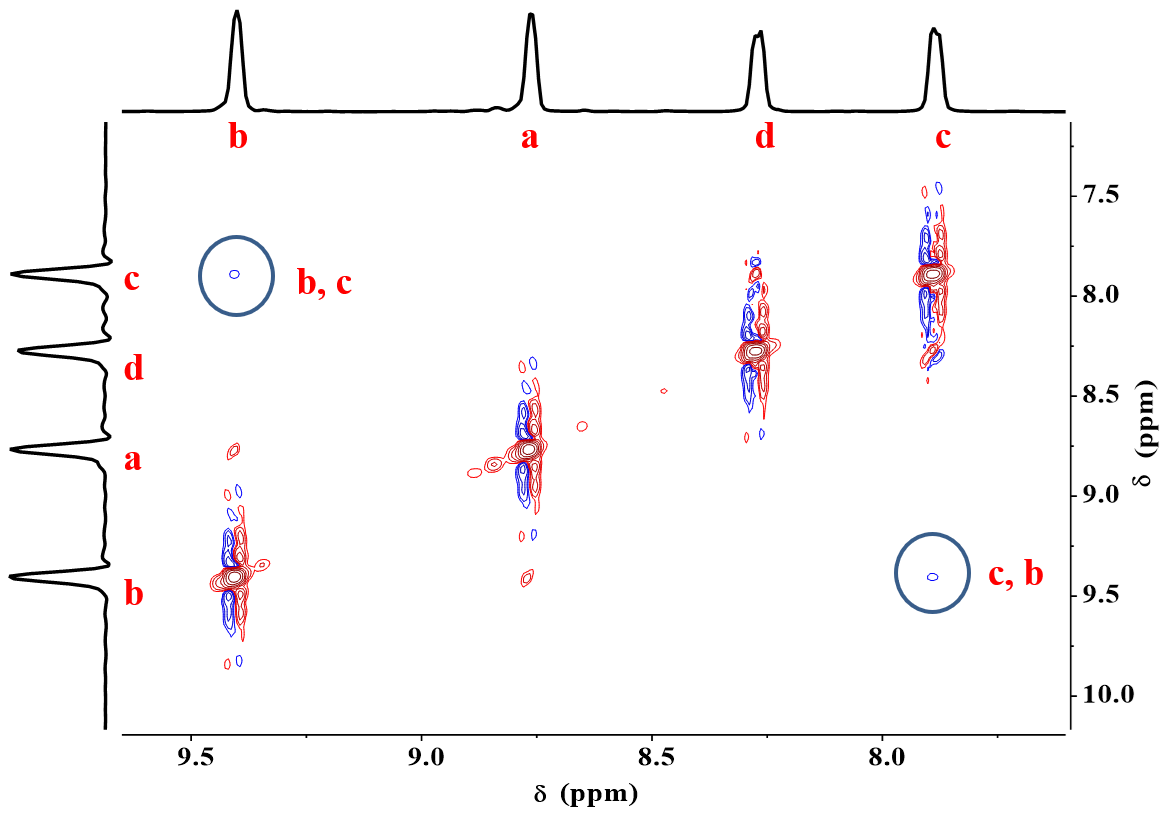
 Figure S2: 2D-NOESY correlation of DPV-COOH.


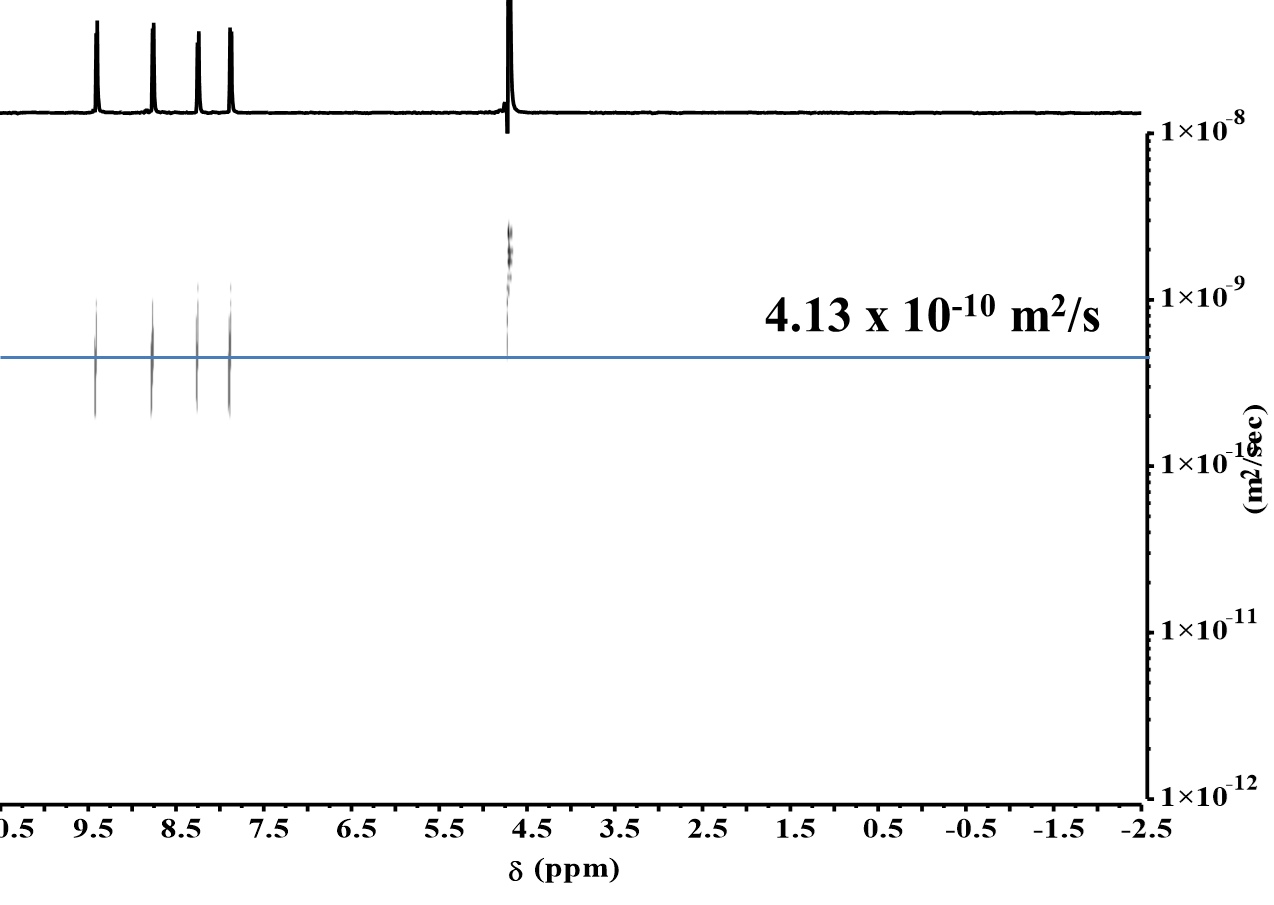


Figure S3: DOSY correlation of DPV-COOH.


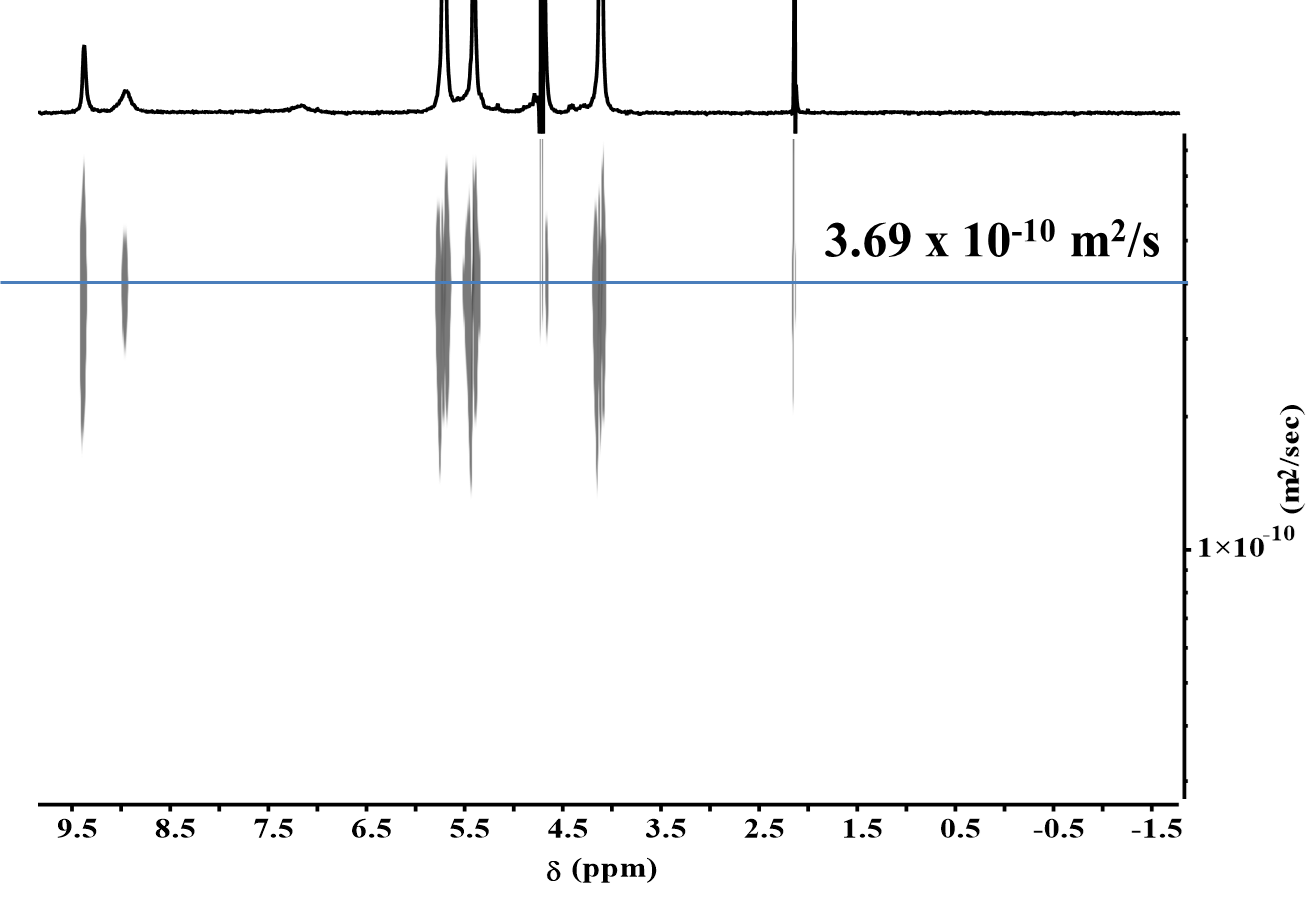


Figure S4: DOSY correlation of DPV-COOH/2 eq CB[8].


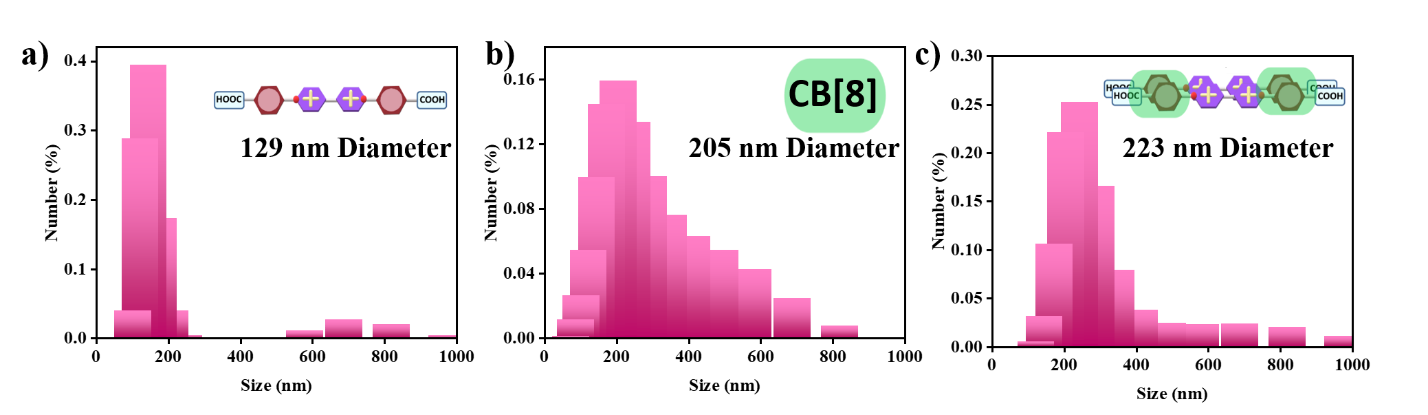


Figure S5: DLS data of a) DPV-COOH, b) CB[8] and c) 2:2 DPV-COOH/CB[8] quaternary complex.


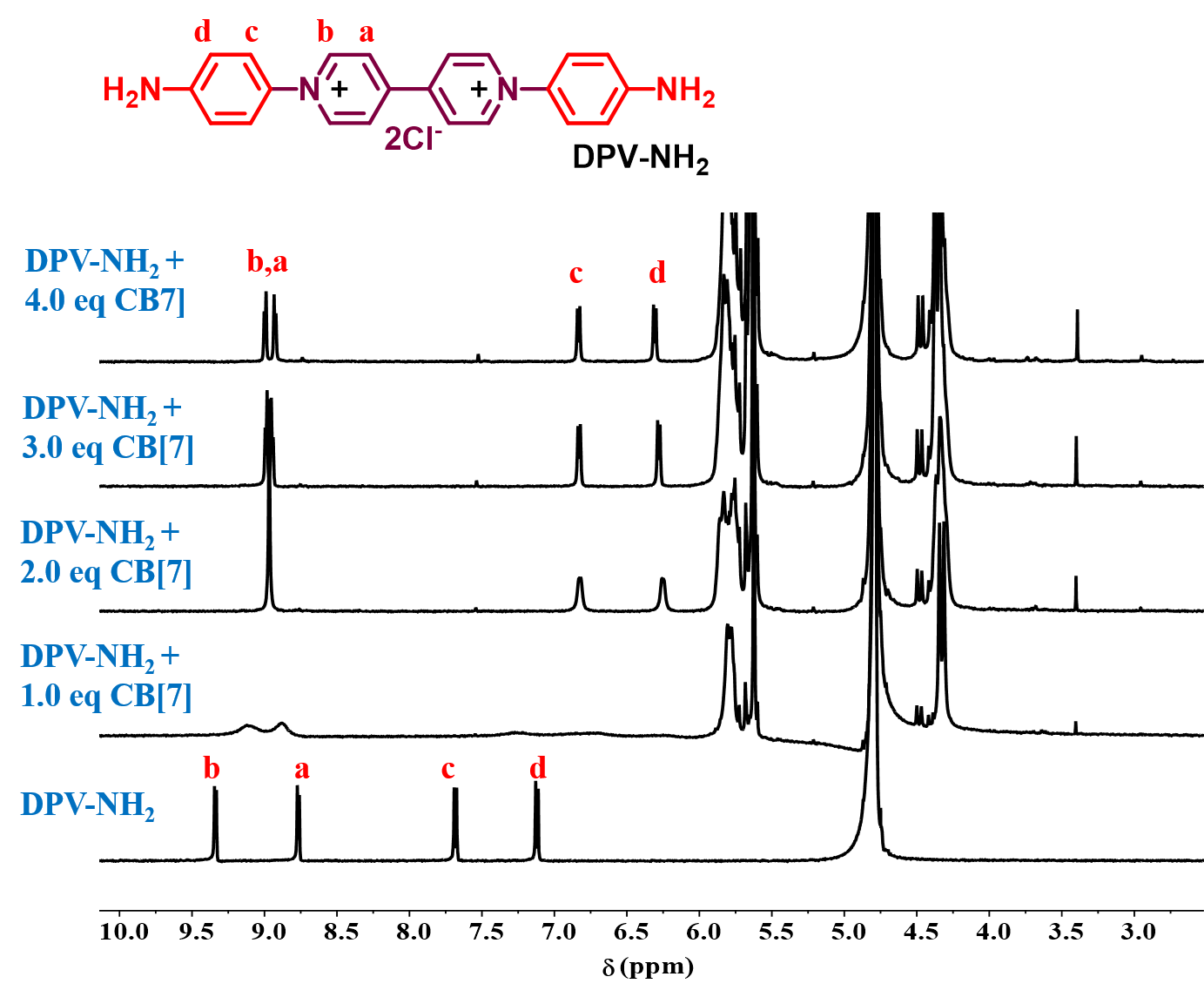
 Figure S6: ^1^H NMR spectra of DPV-NH_2_ with variable concentrations of CB[7] (D_2_O).


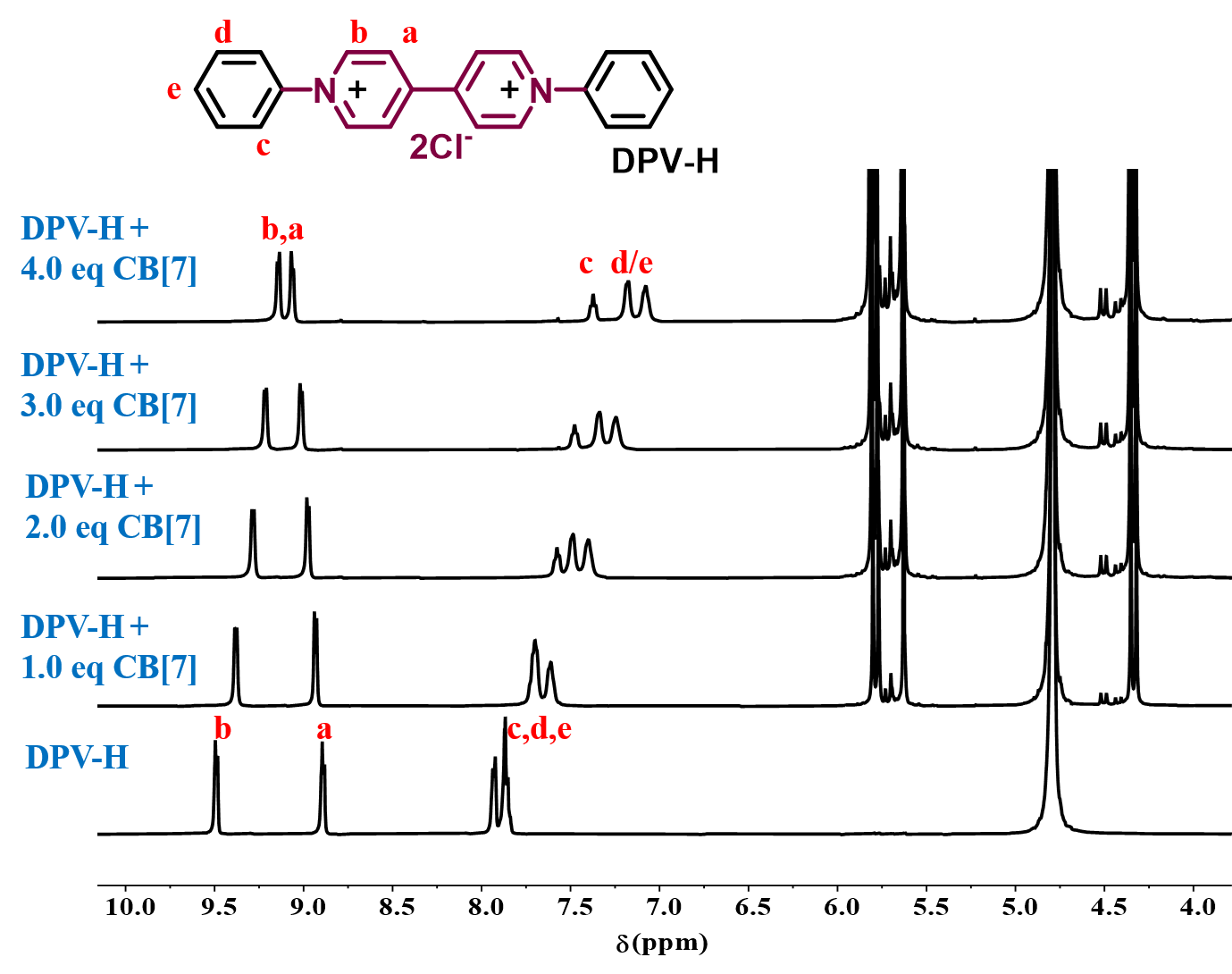
 Figure S7: ^1^H NMR spectra of DPV-H with variable concentrations of CB[7] (D_2_O).


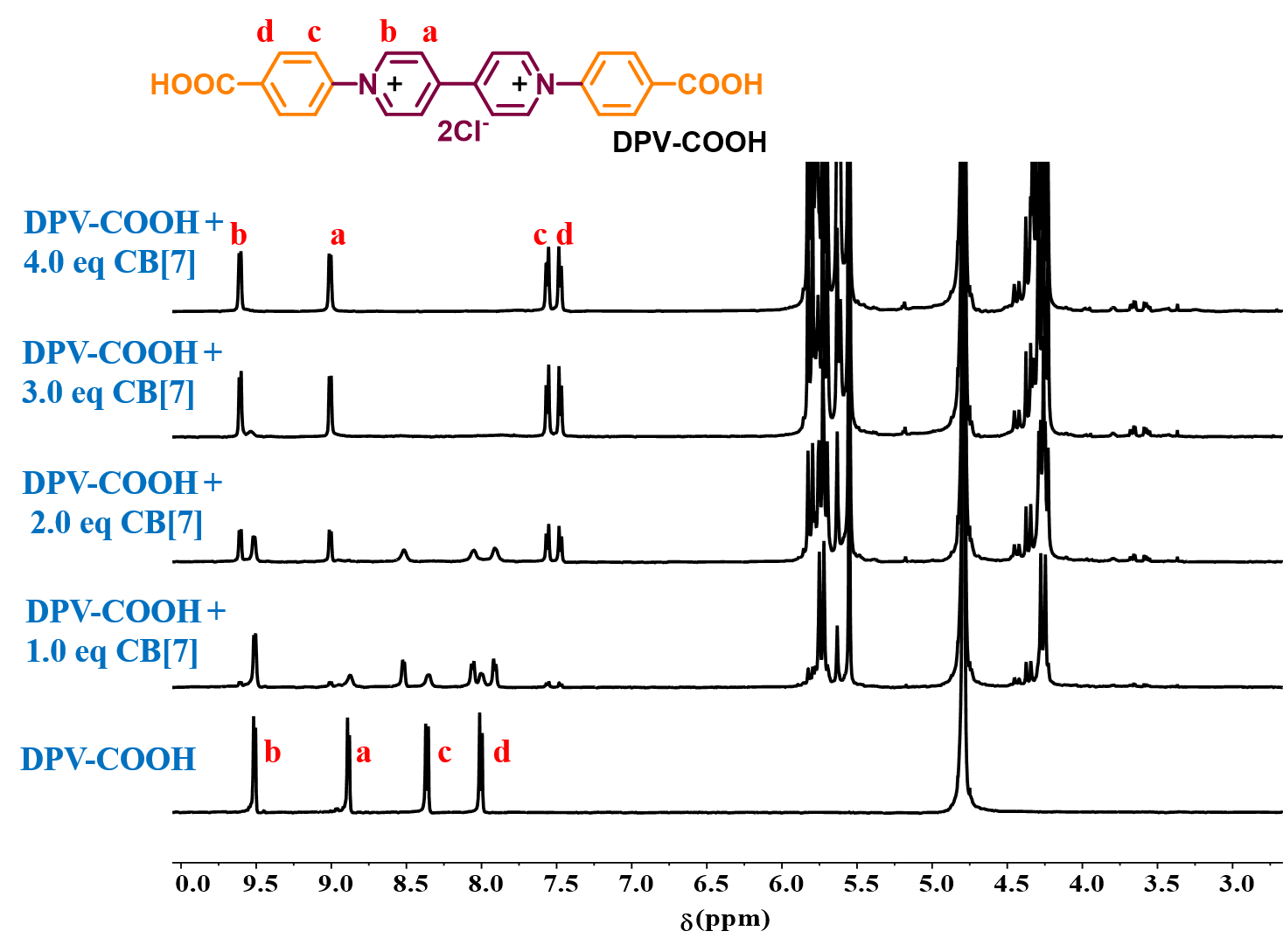
 Figure S8: ^1^H NMR spectra of DPV-COOH with variable concentrations of CB[7] (D_2_O).


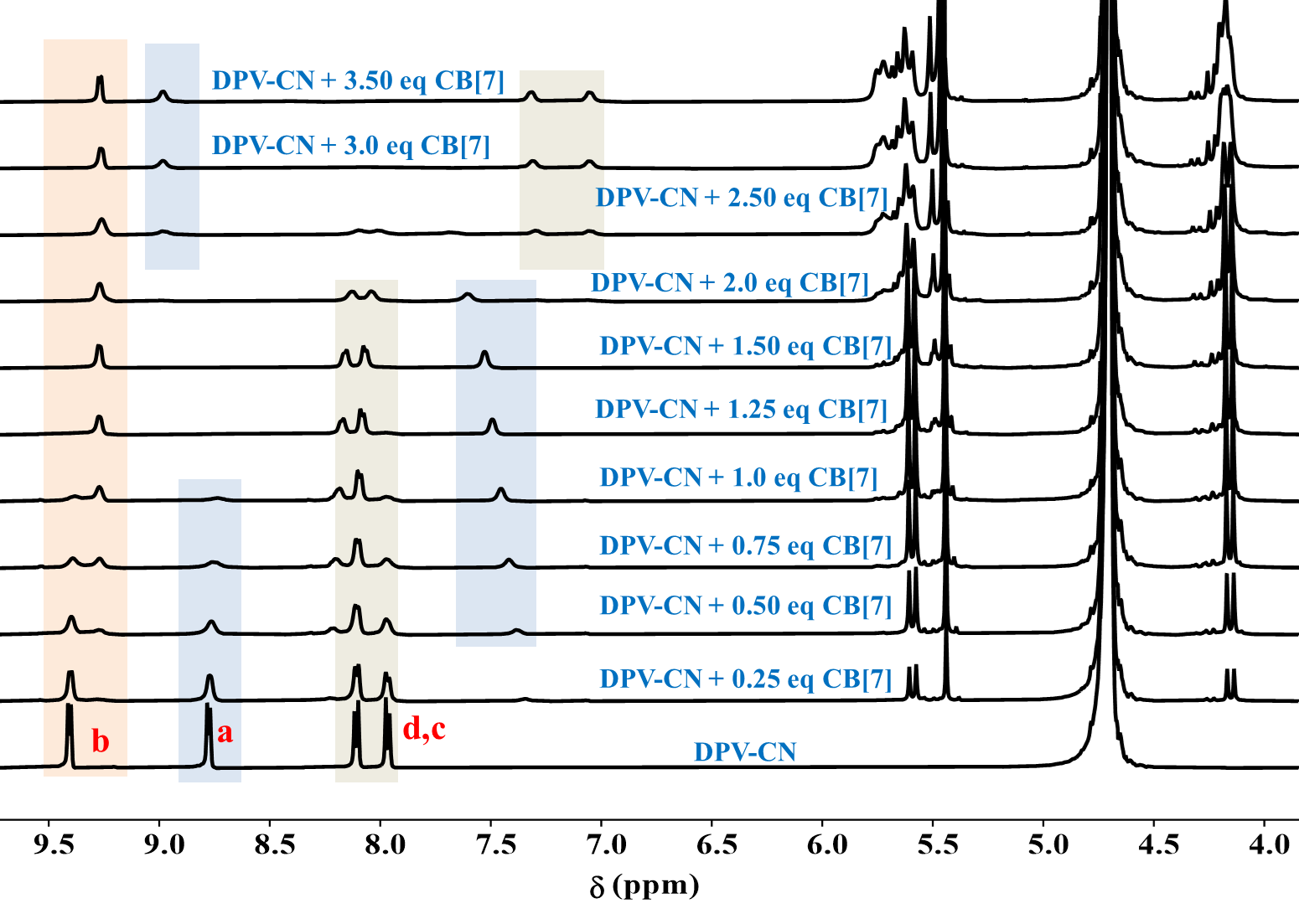


Figure S9: ^1^H NMR spectra of DPV-CN with variable concentrations of CB[7] (D_2_O).
